# Neglected and Underutilized Crop Species: The Key to Improving Dietary Diversity and Fighting Hunger and Malnutrition in Asia and the Pacific

**DOI:** 10.3389/fnut.2020.593711

**Published:** 2020-11-19

**Authors:** Xuan Li, Rashmi Yadav, Kadambot H. M. Siddique

**Affiliations:** ^1^Food and Agriculture Organization of the United Nations (FAO), Regional Office for Asia and the Pacific, Bangkok, Thailand; ^2^ICAR-National Bureau of Plant Genetic Resources Pusa Campus, New Delhi, India; ^3^The UWA Institute of Agriculture, The University of Western Australia (UWA), Perth, WA, Australia

**Keywords:** Asia pacific region, food security and nutrition, future smart food, neglected and underutilized species, malnutrition, dietary diversity, healthy diet, sustainable development goals

## Abstract

Asia continues to suffer from a high prevalence of malnutrition. Persistent malnutrition can be attributed to low dietary diversity, together with low production diversity. Dietary diversity represents a more healthy, balanced, and diverse diet, which ensures nutrient adequacy. The principle of dietary diversity is affirmed in all national food-based dietary guidelines. Food-based approaches that address malnutrition, especially micronutrient deficiencies, are embedded in evidence-based healthy diet patterns; however, they are disconnected from the current agricultural production system. Promising neglected and underutilized species (NUS) that are nutrient-dense, climate-resilient, profitable, and locally available/adaptable are fundamental to improving dietary and production diversity. The Future Smart Food Initiative, led by FAO's Regional Initiative on Zero Hunger, aims to harness the enormous benefits of NUS in the fight against hunger and malnutrition. Recognizing that NUS covers crops, livestock, fisheries and aquaculture, and forests, the FAO has set crops as an entry point for NUS to address hunger and malnutrition.

## Issue: High Prevalence of Malnutrition in Asia

**Figure 1:**
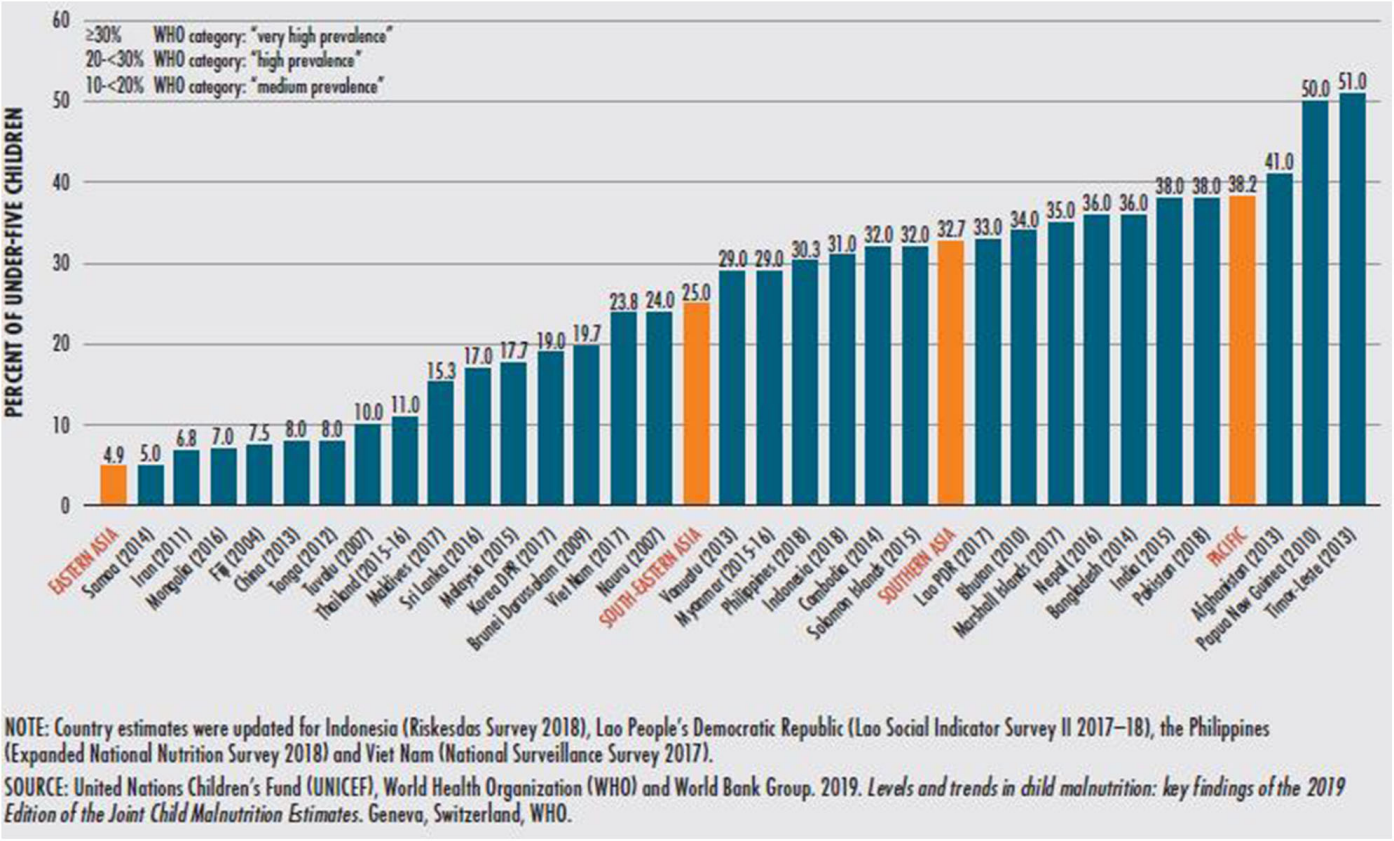
Prevalence of stunting in children under 5 years of age in the Asia Pacific region, by country, latest available year.

The 17 sustainable development goals (SDGs) adopted by the General Assembly of the United Nations in 2015 cover a broad range of global issues (1). There is a strong call to end hunger and malnutrition by 2030, especially in the second SDG2. Other related SDGs include SDG3 (good health and well-being), SDG12 (responsible consumption and production), and SDG15 (life on land). Although substantial advances have been made, ending hunger and malnutrition remain a major concern in the Asia Pacific region. In 2018, the region had an estimated 479 million undernourished people, being 58% of the worldwide total (2). Countries in the region are still facing a high prevalence of undernutrition, especially chronic undernutrition. Chronic undernutrition is measured by stunting (low height for age) and is due to the persistent inability to meet minimum micronutrient and macronutrient requirements, or the frequent recurrence of acute malnutrition episodes, or a combination of both. Stunting, wasting, and underweight are important indicators of child undernutrition. The region has a high prevalence of stunting and wasting, with an estimated 77.2 million children under 5 years of age suffering from stunting and 32.5 million suffering from wasting in 2018 (2). In South Asia, an estimated 58 million children suffer from stunted growth; the prevalence of stunting is medium to very high (over 40%) in most developing countries in the region [apart from China, Fiji, Iran (Islamic Republic of), Mongolia, Samoa, and Tonga] (Figure 1) (2).

**Figure 2:**
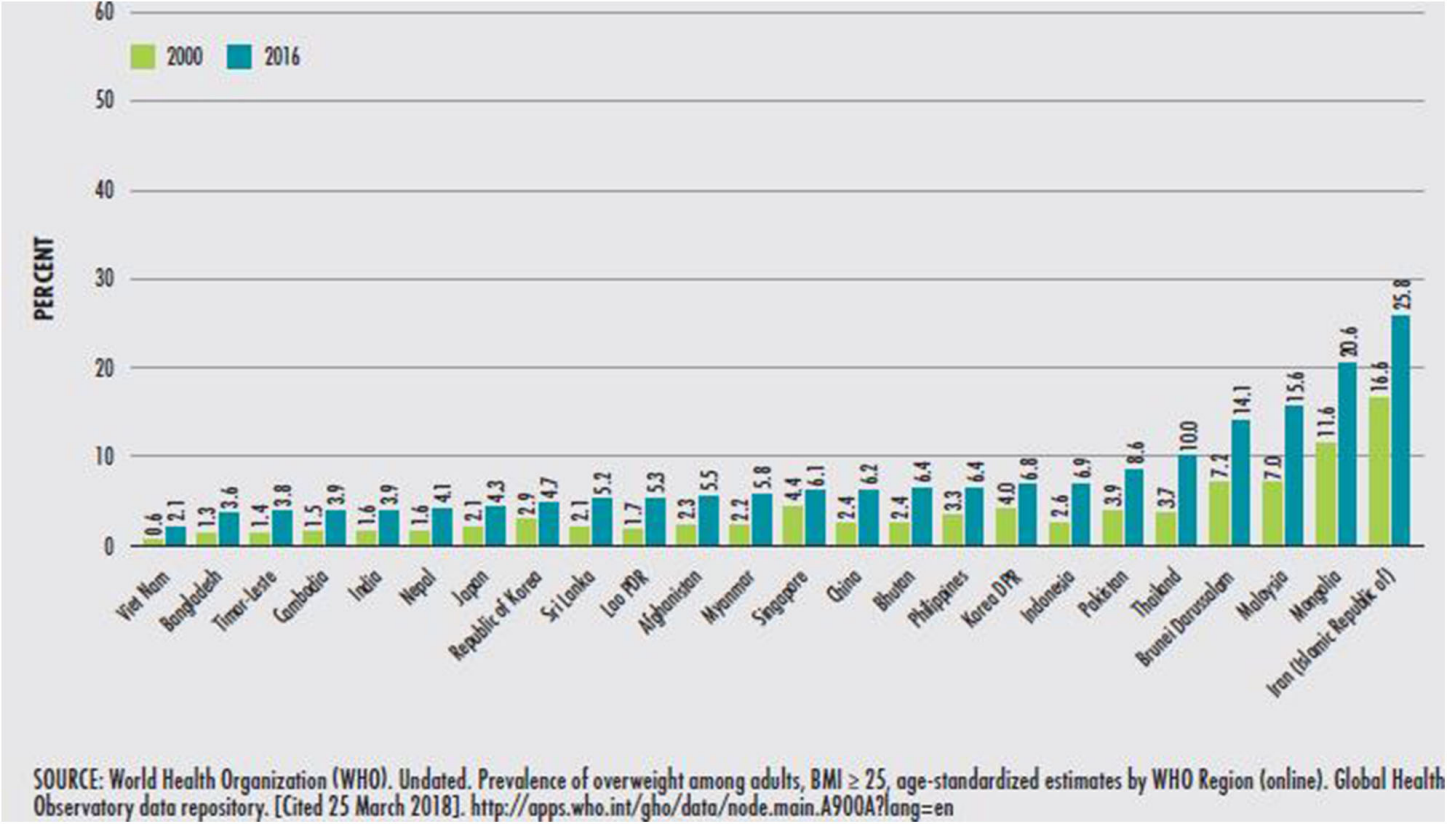
Trends in the prevalence of adult obesity in Asia, by country, 2000 and 2016.

There is an increasing incidence of adult obesity throughout the Asia Pacific region (Figure 2) (2). Overweight and obesity are significant risk factors for all age groups for many diseases, including non-communicable diseases (NCDs), such as diabetes, hypertension, cardiovascular diseases, certain cancers, obstructive sleep apnea, osteoarthritis, respiratory diseases, and diabetes. Worldwide, NCDs are the leading cause of death; in the Asia Pacific region, premature NCD deaths (death before age 70) are high (2). The prevalence of obesity-related diseases, including NCDs, has increased in many countries in the region, particularly the Pacific Islands (2).

High rates of micronutrient deficiency are being observed in many countries in the region. For instance, the prevalence of anemia (iron deficiency) in most ASEAN countries is alarming, amounting to more than 40% for children under 5 years. Countries suffering from severe micronutrient deficiencies (HHI > 25) include Nepal, India, Bangladesh, Bhutan, and Lao PDR (3). In Myanmar, the prevalence of anemia in children under 5 years and reproductive and pregnant women is 57.4, 46.6, and 54.0%, respectively. The prevalence of anemia in Cambodia, Lao PDR, and Nepal ranges from 40–55%.

## Why is Malnutrition High?

### Low Dietary Diversity

Why is malnutrition so prevalent in Asia when most countries are increasing food production to feed the growing population? Malnutrition is generally the result of an inadequate diet, with insufficient nutrients, minerals, and vitamins for the growth and maintenance of a healthy body. The inadequate consumption of food (quantity and quality) or inadequate childcare and feeding practices cause childhood stunting, wasting, and overweight (2).

Dietary diversity in children is positively correlated with the mean micronutrient adequacy of the diet, that is, adequate nutrients for growth and development (4). Minimum dietary diversity (MDD) is a measure of the dietary quality and feeding practices of children (5). An infant or young child is considered to have reached MDD if he/she has consumed food from five of the eight food groups in the previous 24 h. The eight food groups are: (1) grains, roots, and tubers; (2) legumes and nuts; (3) dairy products; (4) flesh foods, including meat, poultry, and fish; (5) eggs; (6) vitamin A-rich fruits and vegetables; (7) other fruits and vegetables; (8) breastmilk (2). Low dietary diversity usually comprises a high consumption of cereals, mainly rice, and relatively low consumption of vegetables, fruits, and pulses.

In the Asia Pacific region, dietary quality and diversity are suboptimal, particularly among infants and young children, with fewer than 50% of children fulfilling the MDD in 15 of the 20 countries. Only 20 and 21% of children achieve MDD in India and Myanmar, respectively. In most countries in the region, less than half of all very young children (aged 6–23 months) meet the minimum standards of dietary diversity; hence, the high prevalence of stunting and wasting among children under five years of age (2).

### Evidence

**Figure 3:**
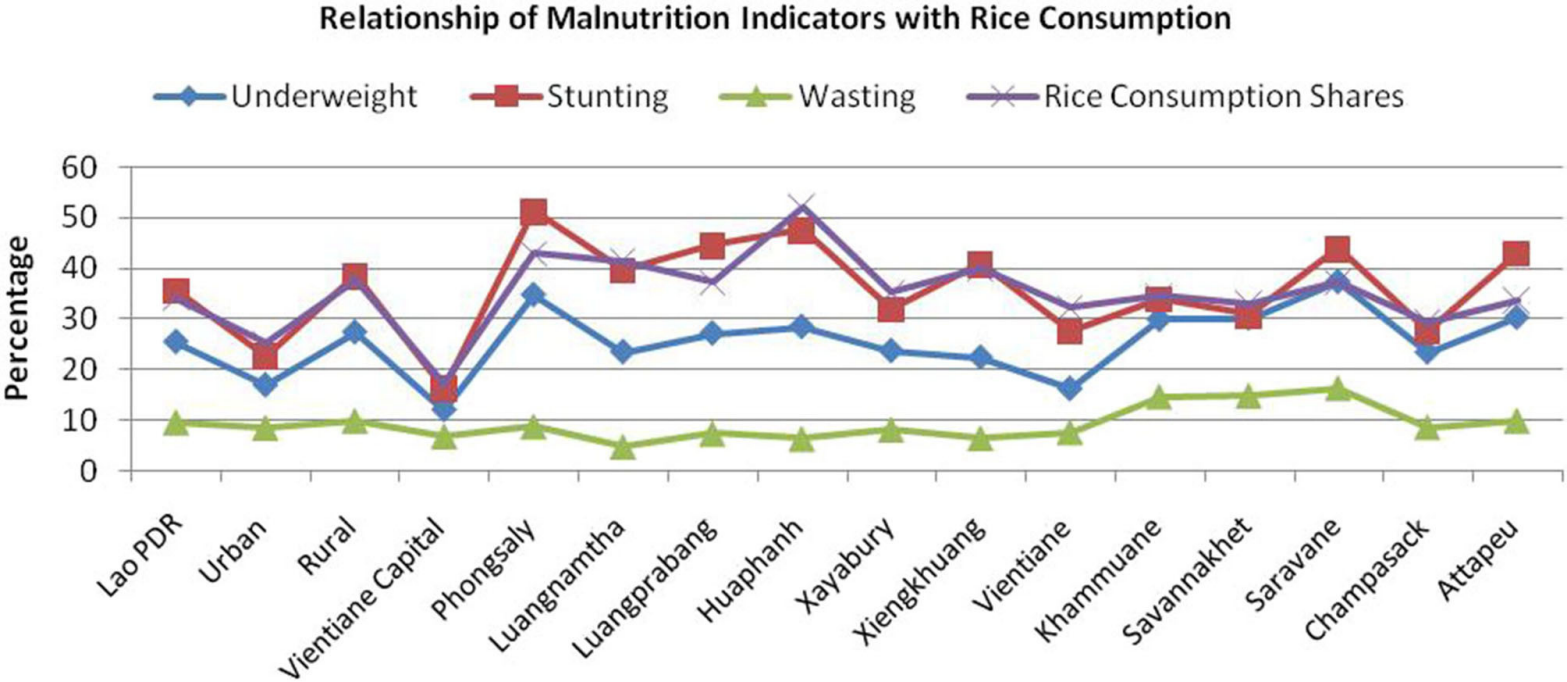
Relationship between rice consumption and malnutrition indicators for Laos and sub-regions 2014.

What is the relationship between malnutrition and dietary pattern? The evidence suggests that high rice consumption areas tend to have high levels of stunting and underweight, particularly in rural areas. In Laos, rice consumption is positively correlated with stunting (Figure 3). The Phongsaly and Huaphanh provinces—where rice constitutes 43 and 52.2% of the diet, respectively—have high levels of all three malnutrition indicators (stunting, wasting, and underweight) (6).

In Myanmar and sub-regions, strong negative relationships exist between stunting and dietary energy consumption and micronutrient intakes. With high levels of dietary energy consumption, stunting levels are low. Similar relationships exist for protein and fat intakes, indicating that stunting is highly correlated with dietary quantity and quality. A reduction in stunting rates requires increased dietary energy consumption, accompanied by more diversified food products.

Anemia has a strong negative correlation with dietary energy consumption and macronutrient intake. Low dietary energy consumption is linked with high anemia among urban populations. Protein-rich foods also help to reduce anemia levels among children. Foods rich in proteins and fats, coupled with an overall increase in consumption, will help combat diseases and improve children's health.

### How to Ensure Dietary Diversity?

**Figure 4:**
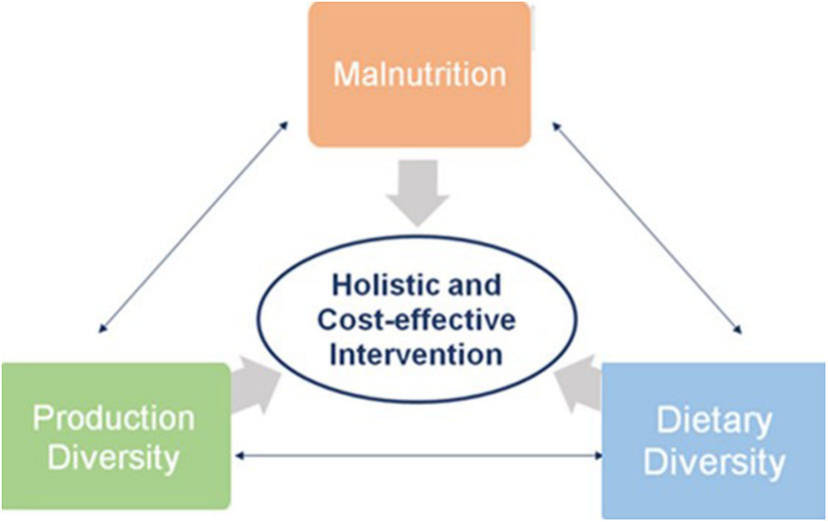
Features of agriculture and food systems Source: Li and Siddique (6).

Agricultural diversification is a formidable tool for achieving Zero Hunger (7). However, current agriculture and food systems have (1) have limited production diversity, resulting in unbalanced diets and, thus, malnutrition. In the Asia Pacific region, only a few staple crops are grown, mainly rice, which form the bulk of people's diets—the lack of dietary diversity fails to deliver wholesome nutrition, as per the recommended nutrition intake (8). The prevalence of rice cultivation in the region is associated with multiple factors: national policies in favor of rice production and consumption; rice varieties from earlier breeding activities that focused on yield resulting in increased energy content and low nutrient density with little consideration of the higher nutritional profiles of indigenous rice varieties; established agronomic practices and environmental conditions for rice cultivation; cultural preference and social recognition for rice as rich people's food; poverty not allowing for diversifying food procurement and thus using the most available; and (2) high-input requirements, such that farming is vulnerable to environmental stresses (9). According to the FAO (10), the reliance on only a few crops negatively affects ecosystems, food diversity, and health. Food monotony increases the risk of micronutrient deficiencies (10). Dietary diversity is a cost-effective, affordable and sustainable way to minimize hunger and malnutrition, and production diversity facilitates the supply of nutritious and diversified food and addresses the effects of climate change (Figure 4) (6).

## Addressing Malnutrition: A Healthy Diet

### What Is a Healthy Diet?

Healthy diets have an optimal caloric intake comprising a diversity of plant-based foods, low amounts of animal source foods, unsaturated rather than saturated fats, and limited amounts of refined grains, highly processed foods, and added sugars (11). Generally, a healthy diet provides the right nutrients (energy, protein, fats, fiber, and essential nutrients such as carbohydrates, amino acids, fatty acids, vitamins, minerals, and fluids) in the right balance, with sufficient diversity for healthy growth and reducing the risk of diet-related diseases (2). While humans are omnivores, not herbivores, a healthy diet is often largely plant-based and includes modest amounts of fish, meat, and dairy (11–14).

### What Is the Benefit of a Healthy Diet?

Micronutrient deficiency mostly affects children and women, particularly those of reproductive age. While the most common indicators for malnutrition in children under 5 years of age are stunting, underweight, and wasting, those for women and children (>5 years) are anemia and vitamin A deficiency. Of the world's estimated 7,000 million people, 500 million suffer from protein-energy malnutrition, >1,600 million suffer from iron deficiency, and >200 million suffer from vitamin A insufficiency (15, 16). More than 400,000 children under 5 years are estimated to die each year from zinc deficiency (17). Diet is one of the most important contributors to health but also disease. Inadequate diets have a direct negative impact on the health of individuals, leading to high NCDs and even death. An unhealthy diet is a significant contributor to most NCDs (18). A systematic evaluation of dietary consumption patterns across 195 countries suggested that dietary improvements could prevent one in every five premature deaths globally (19). The WHO estimates that diets low in fruits and vegetables cause 2.7 million deaths each year and about 19% of gastrointestinal cancer, 31% of ischemic heart disease, and 11% of strokes (20). That is, diet-related NCDs are a leading preventable cause of death worldwide. Dietary modifications toward healthy diets are expected to result in significant health benefits, including preventing 19–24% of total deaths among adults (11).

### Healthy Diets in the Real World

The Mediterranean diet and the Japanese diet are two examples of a healthy diet. The Mediterranean diet—mainly incorporating legumes, cereals, fruits and vegetables, olive oil, fish, and moderate consumption of dairy products (mostly cheese and yogurt)—is the traditional way of eating around the Mediterranean basin. The Mediterranean diet emphasizes the consumption of plant-based foods, including fruits, vegetables, beans, nuts, cereals, and other seeds, olive oil as the main source of dietary fat, red meat in moderation, and herbs and spices instead of salt to flavor food. Compared to the “modern Western” diet, the Mediterranean diet contains much higher quantities of unprocessed foods, uses much less red meat, and has a much higher proportion of unsaturated fats (21). The Japanese diet emphasizes the consumption of fish as a major source of protein, vegetables (including daikon radish and sea vegetables), rice, soy (tofu, miso, soy sauce), noodles, fruit, and tea (preferably green). Fish features prominently in Japanese cuisine: Japanese account for only 2% of the global population, but they collectively consume 12% of the world's fish. With its high popularity, Japanese cuisine is often associated with *sushi* (raw fish and rice served with pickled ginger) and *sashimi* (fresh raw seafood that is dipped in soy sauce and *wasabi*) (21). While *sushi* and *sashimi* are originally “made in Japan,” Japanese cuisine has had strong external influences: around 300 BC, the Japanese learned how to cultivate rice from China, as well as the preparation of soy sauce and tofu (important sources of plant protein). The other external influence was Buddhism: a ban on eating meat was promulgated with the arrival of Buddhism in the seventh century. The popularity of *sushi* came about as a result of this ban. While not always strictly observed, for many centuries, eating meat, particularly beef, was unthinkable; the beef-eating habit returned to Japan only in the late nineteenth century.

These two examples show healthy diets developed in vastly different cultural, climatic, and geographic settings. Both use diverse ingredients linked to people and cultures as much as to their natural environment. Consequently, the Mediterranean and Japanese diets are on UNESCO's World's Intangible Cultural Heritage list (22, 23). Japan and the Mediterranean countries can demonstrate the health effects of their respective healthy diets. Medical research has shown that the Japanese diet has the lowest prevalence of obesity among developed countries—and other chronic diseases, such as osteoporosis, heart ailments, and some cancers (24). Following the Mediterranean diet for several years reduces the risk of developing heart disease, cancer, hypertension, Type 2 diabetes, Parkinson's disease, and Alzheimer's disease (25).

Indeed, the Japanese have one of the longest average life expectancy in the world-−87.45 years for women and 81.41 years for men in 2019—according to the Japanese Ministry of Health, Labor, and Welfare (26). Japanese women outstrip all competitors in life expectancy, including their American counterparts, who can expect to live up to 81 years (76 years for American men). The same holds for developed countries in the Mediterranean: women in Italy and Spain have a life expectancy of 85 years, while the figure is 83 years in Germany. Both Italian and Japanese men have a life expectancy of 80 years (27).

### Food-Based Dietary Guidelines

A healthy diet is the pillar of well-being throughout a person's life. Policies that aim to prevent malnutrition, primarily by ensuring healthy diets for children to prevent stunting and obesity, are more effective than those aiming to reduce malnutrition (2). Unfortunately, there is no one-size-fits-all healthy diet. A healthy diet must be affordable, based on locally available foodstuffs, and meet cultural preferences (28). Since the First International Conference on Nutrition held in 1992, the FAO together with WHO has worked with governments on national food-based dietary guidelines: short, science-based, positive messages on national healthy eating and lifestyles. National governments use similar approaches. For example, the US Government regularly publishes *Dietary Guidelines for Americans* to show how individuals can have a healthy diet following updated scientific evidence. It is the role of governments and public agencies—rather than special interest groups—to provide unbiased information on what constitutes a healthy diet. The WHO Global Strategy on Diet, Physical Activity and Health (29) and the Commission on Ending Childhood Obesity (30) provide strategies for improving diets and physical activity patterns at the population level (2).

## NUS: The Key to Ensuring Healthy Diets and Food Security and Nutrition

### What Is NUS?

Agrobiodiversity is essential to sustainable agriculture, of which NUS are key elements. About 30,000 edible plant species have been identified worldwide; of these, more than 7,000 crop species have been cultivated for food (31, 32). Currently, fewer than 150 crop species are commercially cultivated; 103 deliver up to 90% of the calories in the human diet, and only four (rice, wheat, maize, and potato) provide 60% of the human energy supply (33). Thus, tens of thousands of edible plant species are relatively “underutilized” and could be used to increase the world's food requirements (34).

Crops can be divided into two main categories (staple and underutilized). Underutilized crops (also called neglected, minor, orphan, promising, or little-used) are mostly wild or semi-domesticated species adapted to local environments (6). These crops were used as traditional foods for centuries but became increasingly neglected when more productive crops became available in farming systems. NUS face multidimensional challenges ranging from agro-technical, socioeconomic, policy, and institutional perspectives that have resulted in their underutilization. Agricultural modernization, widespread monoculture, and the promotion of high-yielding varieties have marginalized NUS, which play a minor role in current farming and food systems. Culturally, NUS has been stigmatized by the perception as “food of the poor,” creating a disincentive for their production and consumption (6). Politically, governments tend to give priority to rice production, such as the rice self-sufficiency policy. In short, the lack of an environment conducive to the production, processing, marketing, distribution, and consumption of NUS prevented them from being included in current diets.

### What Value Does NUS Bring?

Neglected and underutilized species offer immense opportunities to fight poverty, hunger, and malnutrition, and their incorporation into farming systems could lead to nutrient-dense, climate-resilient, and sustainable agriculture. Neglected and underutilized species have high nutritional value and are a good source of micronutrients, protein, energy, and fiber. Many NUS crops can also be grown on marginal land, intercropped or rotated with staple crops, and easily fit with integrated practices (35, 36). Many NUS can tolerate various stresses, which will not only make production systems more diverse but more sustainable and climate-resilient (6).

#### Reduce Malnutrition

**Figure 5:**
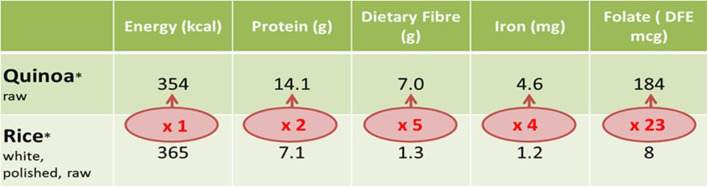
Nutritional comparison of quinoa and rice.

“Hidden hunger”—having enough calories but insufficient vitamins and minerals—is a killer factor affecting both developed and developing countries (37). Hidden hunger is partly due to the reliance on only a few staple crops (38). Since the Green Revolution in the 1960s, agricultural research has focused on increasing crop yields to deliver sufficient food for the world's growing population. Nutritional quality has been less of a concern, despite many people suffering from hunger. While people's standard of living has improved, the effect of diets deficient in essential vitamins and minerals has become apparent in many parts of the world. Neglected and underutilized species have the potential to reverse the trend in hidden hunger. They are often richer in nutrients than their more popular staple crop cousins, with high levels of essential micronutrients (minerals, vitamins) and phytochemicals (such as flavonoids) and good macronutrient profiles (energy, fat, protein, carbohydrates) (39). For example, quinoa is a highly nutritious NUS that came into the limelight in 2014 when the United Nations General Assembly endorsed the International Year of Quinoa. Quinoa has twice as much protein, five times more dietary fiber, four times more iron, and 23 times more folate than rice (Figure 5) (40).

**Figure 6:**
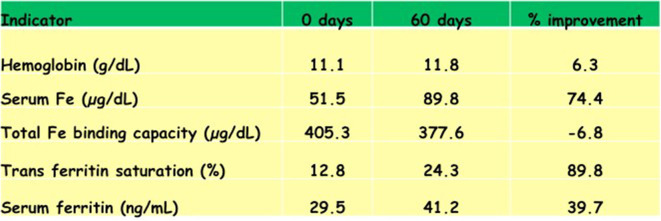
Percent improvement in mildly anemic children (*n* = 33) in Sri Lanka after a 60-day red lentil feeding trial.

Neglected and underutilized species have outstanding health benefits. For example, lentils are rich in micronutrients, with the potential to provide adequate dietary amounts, especially for iron (Fe), zinc (Zn), and selenium (Se) (41). An empirical study by Wijesuriya et al. (42) revealed the impact of an Fe-rich lentil diet on Fe-deficient anemic children in Sri Lanka. The pilot study, involving 33 mildly anemic children (hemoglobin levels = 11 ± 0.8 g/dl), showed a significant improvement in Fe status in the group fed 50 g of red lentils per day for 2 months (Figure 6) (42). These findings indicate that introducing lentils into children's meals, even in the short-term, helps to reduce the prevalence of anemia among children in Sri Lanka by improving their Fe nutritional status and thus has potential in other populations (41). In India, areas that grow lentils had lower anemia rates than those that did not grow lentils. The Indian Government recognized the key role of dietary diversity for preventing nutritional anemia and used food-based approaches to attain adequate dietary iron by encouraging the consumption of micronutrient-rich foods, such as dark green leafy vegetables, lentils, and vitamin-C-rich NUS fruits, which are often available but underutilized by the nutrient-deficient population (6, 43).

Millets also have superior nutritional and health benefits; they are often referred to as “high-energy” cereals, with higher protein, vitamin A and oil contents than maize (44). Vitamin A is often deficient in staple diets, making millets a suitable crop for tackling the nutritional challenges faced by mountain communities (34, 45). Table 1 compares the nutritional value of selected millets and staple crops (46) and shows, for instance, that pearl millet has higher calcium, iron, zinc, riboflavin, and folic acid contents than rice or maize and higher micronutrient contents (excluding calcium) than wheat (47).

**Table 1:** Comparison of nutritional values of selected millets and staple crops.

**Nutrient**	**Selected millets (/100 g)**							**Staple foods (/100 g)**		
	**Pearl millet**	**Sorghum**	**Finger millet**	**Foxtail millet**	**Proso millet**	**Barnyard millet**	**Kodo millet**	**Rice (milled)**	**Maize**	**Wheat flour**
Energy (kcal)	361	349	328	331	341	397	309	345	342	346
Protein (g)	11.6	10.4	7.3	12.3	7.7	6.2	8.3	6.8	11.1	12.1
Fat (g)	5.0	1.9	1.3	4.3	4.7	2.2	1.4	0.4	3.6	1.7
Calcium (mg)	42.0	25.0	344	31.0	17.0	20.0	27.0	10.0	10.0	48.0
Iron (mg)	8.0	4.1	3.9	2.8	9.3	5.0	0.5	3.2	2.3	4.9
Zinc (mg)	3.1	1.6	2.3	2.4	3.7	3.0	0.7	1.4	2.8	2.2
Thiamine (mg)	0.33	0.37	0.42	0.59	0.21	0.33	0.33	0.06	0.42	0.49
Riboflavin (mg)	0.25	0.13	0.19	0.11	0.01	0.10	0.09	0.06	0.10	0.17
Folic acid (mg)	45.5	20	18.3	15.0	9.0	-	23.1	8.0	20	36.6
Fiber (g)	1.2	1.6	3.6	8.0	7.6	9.8	9.0	0.2	2.7	1.2

*Source: NIN (1989)*.

#### Fight Food Insecurity

From a food security perspective, the world's current food system is vulnerable as it relies on a limited range of food items (16). Current farming systems favor monocultures that require high inputs, which facilitate operations but threaten food security. Basing our diet on such a small number of staple crops has serious implications for food security and nutrition (48). The major cultivated crops lack genetic diversity within their gene pools, which leaves agricultural systems exposed to pests and diseases and abiotic stresses (49).

The Great Irish Potato Famine provides an alarming lesson from history. It began in 1845 and lasted for 6 years, killing about two-fifths of the population (over a million people) in Ireland and causing another million to flee the country. The famine was caused by potato blight, a disease that ravaged potato crops throughout Europe. The impact in Ireland was disproportionate, as one-third of the population depended almost entirely on potato for food (50). The marginalized Irish smallholders had cultivated the potato as a staple food since the eighteenth century, as potato yields were much higher per acre than cereals. However, potatoes grown in Ireland were mostly of a single variety, the Irish Lumper. When the disease spread, the lack of genetic variability among the potato plants in Ireland led to devastating effects, while elsewhere in Europe, with more diversity in the varieties of potato being cultivated and/or reliance on a broader range of crops, the effects were much less severe.

Rediscovering neglected crops could reduce the risk of over-reliance on a few major crops. Agricultural sustainability relies on a healthy interaction between agriculture and nature involving three hierarchical levels of genetic diversity: agroecosystems, interspecific diversity (among species), and intraspecific diversity (within species) (51, 52). Marginalizing NUS endangers agrobiodiversity and threatens food system sustainability. Neglected and underutilized species can increase agricultural sustainability by reducing the need for external inputs, such as inorganic fertilizers and pesticides (53). Introducing NUS in a farming system can reduce pest and disease buildup when grown in rotation with main crops. Depending on their characteristics, NUS can also increase soil fertility, prevent soil erosion, reduce evaporation, and suppress weed growth (54).

Neglected and underutilized species are often less demanding of the environment, more resilient to climate change, and more resistant to biotic stresses, thus providing more reliable harvests under unfavorable climatic conditions or on depleted soils (55). For instance, Canahua, an underutilized Andean grain, is remarkably frost tolerant, a key trait of many NUS. Frost-tolerant NUS crops can be grown where high-input major staples fail and are generally more resistant to local pests and diseases. Thus, NUS provide a safety net when the weather turns bad, or external inputs become undesirable as they damage the environment, become unavailable during disasters and emergencies, or become unaffordable due to high prices (6).

Neglected and underutilized crops offer more options for building temporal and spatial diversity into cropping systems. Some NUS have considerable commercial value, such as vegetables and fruits, which can improve household income. Being locally available/adaptable, NUS are accessible and affordable for the local population and therefore contribute to food security and nutrition, livelihood improvements, and cultural diversity (56).

In sum, NUS crops offer superior nutritional value for improving micronutrient deficiencies and addressing NCDs for millions of people. Their resistance to climate change implies that NUS can provide food when other crops fail.

## FAO's Regional Initiative on Zero Hunger on Future Smart Food

Given the multidimensional benefits that NUS offer, and considering that not all NUS are nutrient-dense or climate-resilient, the FAO, in collaboration with national and international partners, under its Regional Initiative on Zero Hunger (RI-ZH), launched a Future Smart Food (FSF) Initiative to support countries in the identification of NUS with high potential to be integrated into agricultural and food systems. The FSF initiative's scope does not include invasive plants and weed species and is focused on crops and their products. Future Smart Food is defined as NUS that are nutrient-dense, climate-resilient, economically viable, and locally available or adaptable (6). Only NUS that met four criteria qualify as FSF, being:

- nutrient-dense (nutrients)
- climate-resilient (e.g., require low inputs, promote climate change resiliency, environmentally friendly by reducing runoff and erosion)
- economically viable (generate income and reduce female drudgery)
- locally available or adaptable (6).

A regional priority-setting exercise for scoping and prioritizing, led by the FAO, supported countries in identifying and prioritizing NUS that qualify as FSF. Considering that NUS covers crop, livestock, fisheries and aquaculture, and forest, FAO started with crop among NUS in the FSF initiative. The FSF initiative started with an interdisciplinary priority-setting exercise comprising three phases in eight countries in Asia: Bhutan, Bangladesh, Cambodia, India (West Bengal), Lao PDR, Myanmar, Nepal, and Vietnam (6):

1. Stage 1: Scoping and identification of NUS (prior to Regional Expert Consultation)
  - Preliminary scoping report on the availability of NUS at the national level
  - Circulation of a preliminary scoping report
  - Review of a preliminary scoping report by international experts designated independently by partner institutions.
2. Stage 2: Validation and prioritization of NUS (during Regional Expert Consultation)
  - Joint validation of preliminary scoping reports from the selected countries
  - Ranking of high-potential NUS according to the four prioritization criteria (i.e., nutrient-dense, climate-resilient, economically viable, and locally available or adaptable)
  - Prioritization of 5–6 NUS crops per country.
3. Stage 3: Mapping
  - Mapping of selected NUS according to their geographical availability/prominence using geographic information system
  - Preparation of GIS reports on selected crops by country.

The regional priority-setting exercise targeted the following food crops groups: (a) cereals, (b) roots and tubers, (c) nuts and pulses, (d) horticulture, and (e) others. Those NUS present in the national gene banks were considered for the exercise. The four FSF prioritization criteria were adapted as (a) nutritional benefits, (b) agricultural sustainability, (c) ecological sustainability, and (d) socioeconomic sustainability, with each criterion further broken down into a series of parameters (e.g., water requirement, drought tolerance, area under cultivation), requiring experts to provide an aggregated dataset on NUS related to each criterion (6). The FSF initiative also established the principle of country ownership. The NUS scoping and prioritization results are owned by the participating country. Considering that NUS is contingent on the local context of each country, a species considered as NUS in one country may not be in another country (6).

**Figure 7:**
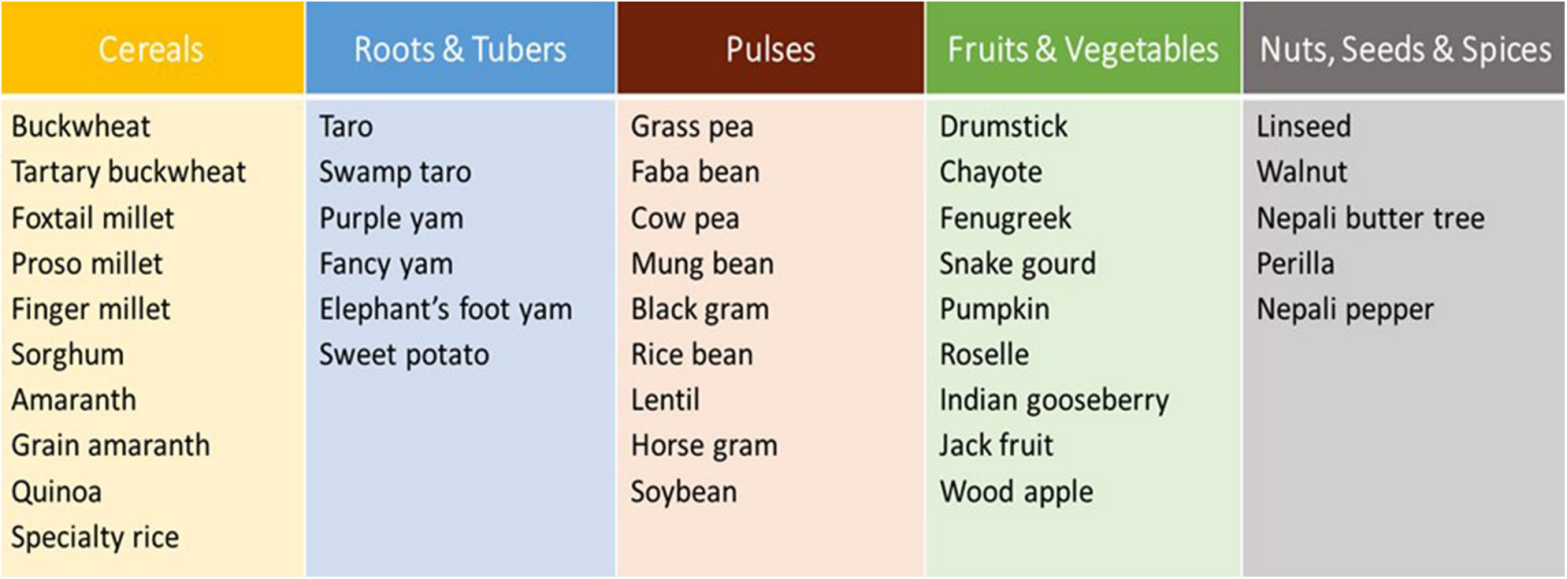
Selected future smart foods in eight countries in Asia Source: Li and Siddique (6).

Applying the same methodology as above, each country nominated national experts by the Ministry of Agriculture and prepared the national scoping reports on promising NUS based on the expertise required as per the guidelines for preparing a national report by the FAO. The NUS scoping and prioritization exercises were entirely country-driven, and the resulting NUS priority lists were determined by a multidisciplinary review of scientific data and specific conditions of the participating country. At the end of the exercise, 39 FSFs were selected and prioritized by the eight countries (Figure 7). All chosen FSFs have the potential to transform current conventional agricultural practices into more sustainable, nutrient-sensitive, and climate-resilient agriculture systems (6).

The integration of FSFs into farming systems has promise for transforming the current agricultural and food system into a more sustainable, nutrient-sensitive, and climate-resilient system (6). While over-dependency on rice with less nutritional properties in comparison to FSF such as quinoa and pulses leads to insufficient intake of nutrient-rich foods (57–60), noting rice-based diet is dominated in Asia culturally, it is culturally more acceptable, technically sound, environmentally mutual beneficial to *integrate* FSF into rice-based production system in Asia, rather than *replace* FSF with rice. The selected FSFs are adapted to different farming systems and agro-ecological zones in the region. Table 2 lists some examples of prioritized FSFs that could be integrated into mountain agriculture and food systems (61).

**Table 2:** FSF examples for mountain areas.

**FSF**	**Image**	**Nutritional and climate-resilient traits**	**Country**
Lentil	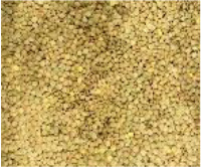	• Second-highest ratio of protein • Huge potential to be grown as a winter crop in warm temperate and subtropical zones	Bhutan, India
Buckwheat	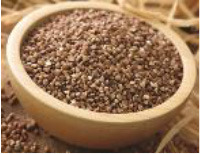	• Rich in iron and zinc—deficiencies of which are a major cause of hidden hunger • Cultivated from alpine to subtropical regions	Bhutan
Mung bean	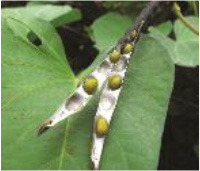	• High in protein, resistant starch, and dietary fiber • Short growing cycle, increased adaptability, drought tolerant	Bangladesh, Nepal, and Viet Nam
Taro	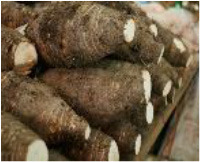	• Rich in carbohydrates and high levels of calcium and vitamin A • Cultivatable in a wide range of areas; multipurpose vegetable with high market value	Bangladesh, Cambodia, Lao PDR, Nepal, Viet Nam, and India
Drumstick	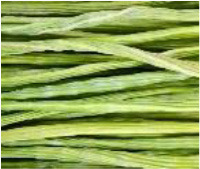	• Rich in calcium, potassium, vitamin A, vitamin C, and protein • Popular vegetable with medicinal value • Powerful anti-inflammatory and antioxidant properties • Fast-growing, drought-resistant	Bhutan, Cambodia, Myanmar, Nepal, Viet Nam, Bhutan, India, Lao PDR
Quinoa	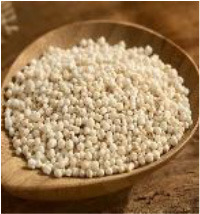	• Rich in fiber, antioxidants, protein, iron, and zinc • Climate-resilient; adapts well to various altitudes	Bhutan, Nepal and Lao DPR
Foxtail millet	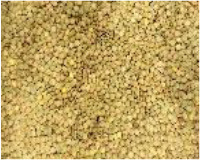	• Helps to control blood sugar levels and reduces the risk of heart attack • Climate-resilient crop; grows in a wide range of agro-climatic conditions • Suitable for cultivation in marginal soils of char land	Bangladesh and India

*Adapted from Li and Siddique (6)*.

## Way Forward

Future smart foods can play a key role in transforming agriculture and food systems into diversified, nutrition-sensitive, and climate-resilient if they are mainstreamed into farming systems. Prioritization of NUS as FSFs is the first step. Moving forward, the FSF value chain must be promoted from production, post-harvest and processing, marketing to consumers, and all stages of the food system are connected to minimize transaction costs (61). Future smart foods need to be produced and marketed in large quantities to guarantee economies of scale and access to upmarket outlets (groceries, supermarkets, export markets). It involves addressing many constraints: from better management of genetic resources, production, processing, and marketing of crops to educating consumers on the nutritional and healthy benefits of FSF.

**Figure 8:**
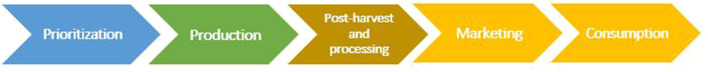
Development stages of food systems for future smart foods Source: FAO (62).

A holistic food systems approach for FSF has been developed as follows (see also Figure 8):

1. Prioritization: identify and prioritize NUS as potential FSFs.
2. Production: increase the production of targeted mountain FSFs in farming systems adaptable to various agro-ecological zones.
3. Processing: improve the efficiency of post-harvest and processing of FSFs.
4. Marketing: promote the distribution and marketing of FSFs.
5. Consumption: increase the demand for FSFs among consumers by increasing awareness and knowledge on their multidimensional benefits, including nutritional value (61).

Improving value chains for FSFs is critical, which will contribute to increased farmer income and agricultural diversification. Recent examples for the re-discovery of NUS include the growing markets for quinoa, growing sales of farro (an ancient wheat variety, often translated as spelt or emmer) in Italy, and the domestication of tropical fruit trees, such as *Dacryodes edulis* (sometimes called bush butter tree), in West Africa.

Creating an enabling environment for promoting FSF production, marketing, and consumption is crucial. Governments need to tap into the immense potential of alternative NUS crops. Governments can create incentives or remove disincentives, upgrade basic infrastructure to facilitate market access and reduce transaction costs, and improve licensing and legal frameworks to promote FSF and encourage agricultural diversification. Public policies promoting FSF as components of sustainable diets could encourage their use. Incentives can support farmers to grow and conserve NUS on-farm and *ex-situ*. Governments should mainstream FSF best practices, methods, and tools into routine operations, particularly for the rural poor, who suffer the most from production and nutrition gaps, shocks, and uncertainties (63). Traditional food systems in Asia have developed over hundreds of years, featuring an abundance of nutritionally dense and climate-resilient foods: promoting these alternative options offers increased yield potential and an opportunity to diversify dietary patterns and generate income for the rural poor (6).

## Conclusion

While substantial progress has been made, countries in the Asia Pacific region face a high prevalence of hunger and all forms of malnutrition. Why? One of the main causes of malnutrition is an inadequate diet, with insufficient nutrients, minerals, and vitamins to grow and maintain a healthy body. Poor dietary diversity leads to an inadequate diet in terms of quality and hence malnutrition. Dietary diversity is alarmingly low among young children in the Asia Pacific region. Policies aimed at preventing malnutrition through healthy diets are most effective. Unfortunately, there is no one-size-fits-all healthy diet—healthy diets must be affordable, based on locally available foodstuffs, and meet cultural preferences.

Neglected and underutilized species offer enormous opportunities for fighting poverty, hunger, and malnutrition. Many NUS have superior nutritional values for improving micronutrient deficiencies. Many NUS can tolerate various stresses, which would not only make production systems more diverse but also more sustainable and climate-resilient. Their resistance to climate change implies that NUS can provide food when other crops fail. To identify NUS that are nutrient-dense, climate-resilient, economically viable, and locally available or adaptable, the FAO launched a Future Smart Food Initiative to support countries in identifying NUS with high potential for integration into agricultural systems. Consequently, 39 FSFs were selected and prioritized by the countries as the first step, all of which could transform current conventional agricultural practices into more sustainable, nutrition-sensitive, and climate-resilient agriculture systems. Moving forward, future endeavors should promote FSFs in terms of production, post-harvest and processing, marketing, and consumption.

## Author Contributions

XL and KS conceived the idea and returned the first draft. All authors contributed subsequent revisions of manuscript.

## Conflict of Interest

The authors declare that the research was conducted in the absence of any commercial or financial relationships that could be construed as a potential conflict of interest.
